# Single-cell analysis reveals IGF-1 potentiation of inhibition of the TGF-β/Smad pathway of fibrosis in human keratocytes *in vitro*

**DOI:** 10.1038/srep34373

**Published:** 2016-09-30

**Authors:** Tomislav Sarenac, Martin Trapecar, Lidija Gradisnik, Marjan Slak Rupnik, Dusica Pahor

**Affiliations:** 1 Department of Ophthalmology, University Medical Centre Maribor, Ljubljanska 5, 2000 Maribor, Slovenia; 2 Institute of Physiology, Faculty of Medicine, University of Maribor, Taborska ulica 8, 2000 Maribor, Slovenia; 3 Gladstone Institute of Virology and Immunology, University of California, 1650 Owens Street, 94158 San Francisco, CA, USA; 4 Institute of Physiology, Centre for Physiology and Pharmacology, Medical University of Vienna, Währingerstrasse 13A, 1090 Vienna, Austria

## Abstract

Corneal wound healing is often affected by TGF-β–mediated fibrosis and scar formation. Guided fibrosis with IGF-1 and antifibrotic substances might maintain corneal transparency. Primary human corneal keratocytes under serum-free conditions were used as a model of corneal stromal wounding, with markers of corneal fibrosis and opacity studied under TGF-β2 stimulation. Single-cell imaging flow cytometry was used to determine nuclearization of Smad3, and intracellular fluorescence intensity of Smad7 and the corneal crystallin aldehyde dehydrogenase 3A1. Extracellular matrix proteoglycans keratocan and biglycan were quantified using ELISAs. On the TGF-β2 background, the keratocytes were treated with IGF-1, and suberoylanilidehydroxamic acid (SAHA) or halofuginone ± IGF-1. IGF-1 alone decreased Smad3 nuclearization and increased aldehyde dehydrogenase 3A1 expression, with favorable extracellular matrix proteoglycan composition. SAHA induced higher Smad7 levels and inhibited translocation of Smad3 to the nucleus, also when combined with IGF-1. Immunofluorescence showed that myofibroblast transdifferentiation is attenuated and appearance of fibroblasts is favored by IGF-1 alone and in combination with the antifibrotic substances. The TGF-β/Smad pathway of fibrosis and opacity was inhibited by IGF-1, and further with SAHA in particular, and with halofuginone. IGF-1 is thus a valid aid to antifibrotic treatment, with potential for effective and transparent corneal wound healing.

One of the significant causes of blindness worldwide is reduced corneal transparency due to improper fibrosis after corneal injury or inflammation^1^. The cornea is mostly made of rigid layers that are interspersed with keratocytes, which are small in size and metabolically quiescent. Stromal extracellular matrix (ECM) layers are formed as packed lamellae of arrayed collagen bundles that are aligned and supported by the corneal proteoglycans keratocan^2^, lumican^3^,^4^, decorin^5^, and mimecan^6^. This precise arrangement provides transparency for the visual spectrum of light, and forms the major refractive curvature of the eye^7^. To minimize light-scattering by keratocytes, these contain high levels of intracellular crystallins^8^, which include in particular aldehyde dehydrogenase 3A1 (ALDH3A1)^9^,^10^.

During corneal trauma (e.g., wounding, refractive surgery, infection, chemical burn) the keratocytes are activated by various growth factors, such as transforming growth factor (TGF)-β1 and TGF-β2 and insulin-like growth factor (IGF)-1, which promote the sealing of stromal defects. The individual growth factors and their interactions in the stroma can critically alter the light diffraction and the clinical outcome.

After IGF-1 receptor activation, the keratocytes transdifferentiate to corneal-wound-type fibroblasts^11^, which express Thy-1 at the cell surface^12^ and produce proteoglycans and other components of the ECM. In this way, some of the intracellular transparency diminishes due to down-regulation of the crystallins^11^, although this scenario yields an ECM that is similar in composition to the normal cornea^11^,^13^. In contrast, TGF-β receptor activation leads to corneal fibrosis and a hazed stroma, which results in reduced physiological optical performance^14^,^15^,^16^,^17^,^18^. TGF-β facilitates differentiation of the keratocytes into myofibroblasts^18^,^19^, which are hallmarked by the formation of alpha-smooth muscle actin (α-SMA)^20^ fibers. These cells secrete ECM materials that form non-transparent scaffolds of scar collagen^21^,^22^. With this TGF-β receptor activation, the proteoglycans that are normally scarce in the adult cornea, such as biglycan, are up-regulated^23^,^24^, while the expression of keratocan, which is normally abundant, is down-regulated^25^,^26^,^27^. Furthermore, TGF-β down-regulates crystallin expression, which increases the light scattering of these cells^28^.

Guided fibrosis of keratocytes has therefore gained significant interest in recent years, and several molecular targets related to fibrosis have been identified. The TGF-β/Smad signaling axis is believed to be one of the main TGF-β downstream cascades connected to fibrotic corneal wound healing^18^,^29^,^30^,^31^. The activated TGF-β receptor complex phosphorylates Smad3, which then functions as a nuclear signal transducer^32^. Smad7 is then expressed, which acts as an antagonist of this TGF-β signal transduction, as part of a negative-feedback loop^31^,^33^.

DNA histone acetylation/deacetylation, i.e., epigenetic therapy, has been proposed for the modulation of TGF-β–mediated fibrosis^34^,^35^,^36^. Suberoylanilidehydroxamic acid (SAHA; also known as vorinostat) has been demonstrated to be a potent histone deacetylase (HDAC) inhibitor that is safe for application to the ocular surface^37^. Using rabbits in a corneal haze model, SAHA was validated as an effective inhibitor of post-photorefractive keratectomy corneal haze. Furthermore, *in-vitro* studies on animal and human corneal fibroblast wounding models have shown disruption of TGF-β–mediated fibrotic responses^38^,^39^ and inhibition of Smad2/3 phosphorylation^40^,^41^. SAHA has also been approved for human use for the treatment of cutaneous T-cell lymphoma by the US Food and Drug Administration^42^.

Halofuginone is a natural metabolite from the ‘evergreen hydrangea’ *Dichroa febrifuga* that has been approved for anti-fibrotic therapy in patients with scleroderma by the US Food and Drug Administration^43^, and it has been shown to inhibit the transformation of fibroblasts into myofibroblasts^44^. Halofuginone inhibits the expression of Smad3 and its phosphorylation^45^,^46^, with up-regulation of Smad7^47^ and down-regulation of TGF-β2 receptors^48^.

Although the effects of SAHA and halofuginone on the TGF-β/Smad pathway in keratocytes have been defined to some extent, there remains the need to further optimize wound-healing responses pharmacologically, by augmenting the beneficial aspects of the healing, and inhibiting the associated fibrosis and scarring^49^. Guided fibrosis might inhibit some of the vital roles of TGF-β in wound healing, particularly in terms of cell proliferation^50^. Here, IGF-1 might therefore be a suitable additional growth factor to compensate for these effects^48^,^51^, while it should encourage transdifferentiation of the keratocytes to a more regenerative phenotype–to wound-type corneal fibroblasts^11^.

This concise report presents the effects of the combination of IGF-1 with the antifibrotics SAHA and halofuginone (both approved for human use) on TGF-β2–activated primary human keratocytes as a cell-culture model of corneal stromal wounding. We thus studied TGF-β/Smad signaling and keratocyte proliferation, along with the ECM composition and the corneal crystallin ALDH3A1, which are detrimental for corneal transparency.

## Results

### IGF-1 alone and in combination with SAHA or halofuginone inhibits nuclearization of Smad3 and modulates Smad7 levels

**Figure 1:**
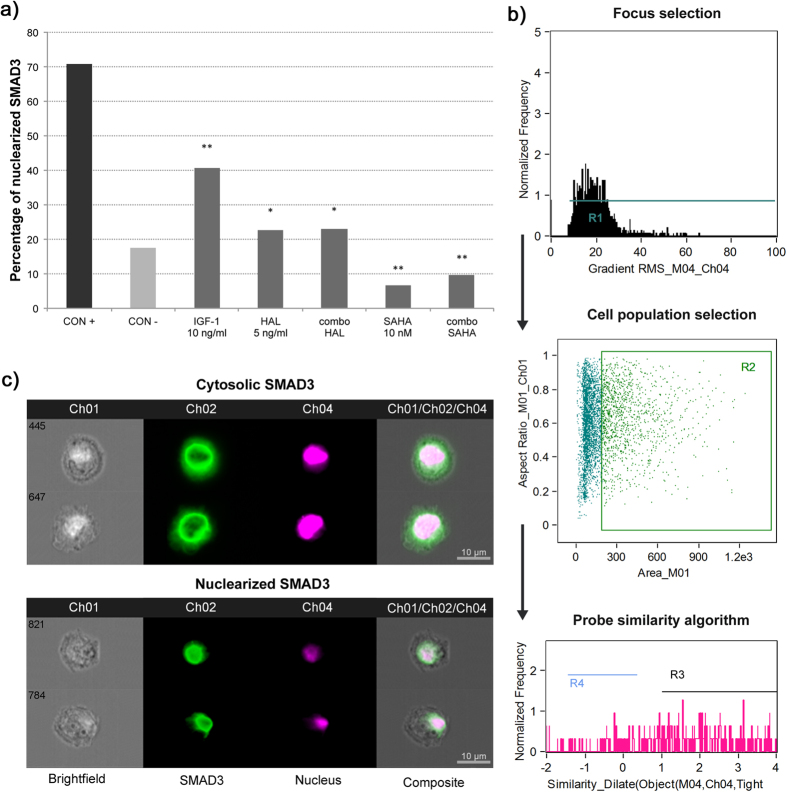
Smad3 translocation to the nucleus, analyzed using imaging flow cytometry. **(a)** Relative levels of Smad3 nuclearization in human keratocytes. *p < 0.001 compared to CON+; **p < 0.001 compared to CON+ and CON− (Chi^2^ tests). **(b)** Workflow diagram of the IDEAS software. First, images of the keratocytes in focus were chosen, and then the relevant cell population was selected, excluding the nonspecific labeling. The probe similarity algorithm for co-localization of the nucleus and Smad3 was then defined. The software calculated the correlation coefficient (Similarity Dilate) of the two probes, plotted as a frequency histogram. R3, keratocytes with Similarity Dilate >1, which implies nuclearization has occurred. **(c)** Representative images of ketatocytes labeled for Smad3 and the nucleus, as revealed by the imaging flow cytometry (green, AlexaFluor® 488, Smad3; purple, 7AAD, nucleus). Upper panel, cytosolic Smad3; lower panel, nuclearized Smad3. CON+, positive control of 10 ng/ml TGF-β2; CON−, negative control for naïve human keratocytes. All of the following conditions also included 10 ng/ml TGF-β2: 10 ng/ml IGF-1; 5 ng/ml halofuginone (HAL); 10 ng/ml IGF-1 plus 5 ng/ml halofuginone (combo HAL); 10 nM SAHA; 10 ng/ml IGF-1 plus 10 nM SAHA (combo SAHA).

For the imaging flow cytometry analysis of Smad3 translocation, we ran an algorithm for co-localization of nuclear and Smad3 probes in the individual naïve human keratocytes treated and analyzed here. As shown in Fig. 1a,b, 70.8% of these keratocytes that were treated with 10 ng/ml TGF-β2 alone (i.e., as positive control; Fig. 1a, CON+) promoted translocation of Smad3 to the nucleus, in contrast to the 17.5% translocation seen for the naïve keratocytes (i.e., as negative control; Fig. 1a, CON−). On this background of 10 ng/ml TGF-β2, treatment of the keratocytes with only 10 ng/ml IGF-1 led to a 30.0% loss in the Smad3 nuclearization compared to the TGF-β2 positive control (40.8% vs. 70.8%, respectively), indicating the anti-TGF-β2 activity of IGF-1 alone (Fig. 1a, IGF-1). The TGF-β2–treated keratocytes were also treated with 10 nM SAHA alone and in combination with 10 ng/ml IGF-1 (Fig. 1a, SAHA, combo SAHA), which showed the lowest levels of Smad3 nuclearization, at 6.6% and 9.7%, respectively; i.e., significantly lower than both the TGF-β2 positive control and the negative control (p < 0.001). The TGF-β2–treated keratocytes were also treated with 5 ng/ml halofuginone alone and in combination with 10 ng/ml IGF-1 (Fig. 1a, HAL, combo HAL), which showed similar levels of Smad3 nuclearization to the negative control (22.6%, 22.9%, respectively), and were significantly lower than the TGF-β2 positive control (p < 0.001).

This imaging flow cytometry analysis of Smad3 nuclearization thus demonstrated high activity for the TGF-β/Smad pathway in the TGF-β2 positive control, and clear inhibition in these treatment groups. Immunoblotting studies of human and canine corneal fibroblasts^40^,^41^ have recently shown SAHA-mediated inhibition of Smad2/3 phosphorylation, which might be the main mechanism for the lower nuclearization of Smad3 observed with these treated keratocytes. Indeed, Smad3 nuclearization was notably decreased by halofuginone, in agreement with a report from Nelson *et al*.^45^.

**Figure 2:**
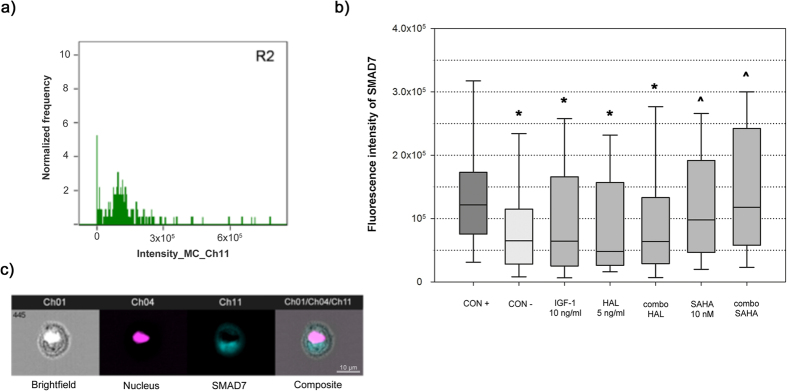
Intracellular Smad7 levels, analyzed using imaging flow cytometry. Data from imaging flow cytometry were first analyzed using the IDEAS software as described in Fig. 1b. (**a**) Frequency histogram of Smad7 fluorescence intensity plotted for each condition. *p < 0.001 compared to CON+; ^p < 0.001 compared to CON− (Mann-Whitney rank sum tests). **(b)** Box and whisker plot of Smad7 median fluorescence intensity. Horizontal line within box, median fluorescence intensity; boundaries of the box, 25^th^ and 75^th^ percentiles; whiskers, boundaries of 5^th^ and 95^th^ percentiles. The median fluorescence intensity can be considered as a relative measure of the intracellular Smad7 levels. **(c)** Representative images of keratocytes labeled for Smad7 and the nucleus, as revealed by the imaging flow cytometry (blue, Cy5, Smad7; purple, 7AAD, nucleus). For details of conditions, see legend to Fig. 1.

Furthermore, imaging flow cytometry analysis was used to determine the median Smad7 fluorescence intensities, which correspond to the intracellular levels of this protein. Significantly higher Smad7 median fluorescence intensity was observed for the TGF-β2 positive control (1.22 × 10^5^, interquartile range, 0.98 × 10^5^; Fig. 2b, CON+) compared to the negative control (Fig. 2b, CON−) and all of the treated groups (p < 0.001), which testifies to the TGF-β2 inhibitory transcriptional feedback loop. The TGF-β2–treated keratocytes that were also treated with IGF-1 or halofuginone or their combination (Fig. 2b, IGF-1, HAL, combo HAL) showed similar Smad7 median fluorescence intensities to the negative control (Fig. 2b, CON−; 0.65 × 10^5^, interquartile range, 0.87 × 10^5^), whereas those also treated with SAHA alone or with the IGF-1 plus SAHA combination (Fig. 2b, SAHA, combo SAHA) showed significantly higher Smad7 median fluorescence intensities (0.98 × 10^5^, interquartile range, 1.45 × 10^5^; 1.18 × 10^5^, interquartile range, 1.84 × 10^5^; respectively; p < 0.001).

This treatment with SAHA resulted in more Smad7, despite the inhibition of Smad3 shown earlier. Halofuginone, on the other hand, did not show any significant increase in the intracellular levels of Smad7 compared to the negative control, which is not in agreement with the report of Saika *et al*.^47^.

### Expression of ALDH3A1 is augmented by the IGF-1 plus SAHA combination

**Figure 3:**
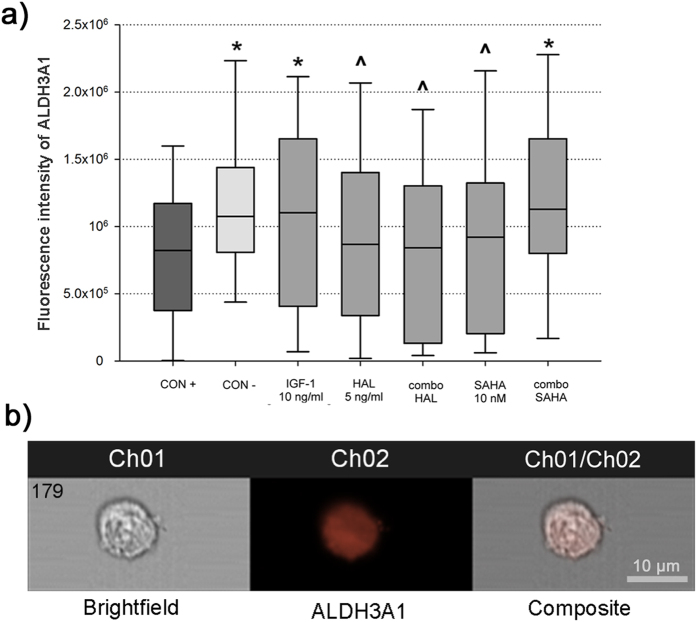
Intracellular ALDH3A1 levels, analyzed using imaging flow cytometry. **(a)** Box and whisker plot of ALDH3A1 fluorescence intensity (see also legend to Fig. 2). The median fluorescence intensity can be considered as a relative measure of intracellular ALDH3A1 levels. *p < 0.001 compared to CON+; ^p < 0.001 compared to CON− (Mann-Whitney rank sum tests). **(b)** Representative images of keratocytes labeled for ALDH3A1, as revealed using imaging flow cytometry (orange, AlexaFluor® 488, ALDH3A1). For details of conditions, see legend to Fig. 1.

The median fluorescence intensity of ALDH3A1 for the negative control (Fig. 3a, CON−) was 24.7% higher (1.01 × 10^6^, interquartile range, 0.67 × 10^6^) compared to the TGF-β2 positive control (Fig. 3a, CON+; 0.81 × 10^6^, interquartile range, 0.80 × 10^6^, p < 0.001). IGF-1 treatment of the TGF-β2–treated keratocytes resulted in a 25.8% increase in ALDH3A1 median fluorescence intensity compared to the TGF-β2 positive control (Fig. 3a, CON+). The lower levels for the TGF-β2 positive control correlates to increased cell light scattering in myofibroblasts, which has previously been coupled with lower levels of corneal crystallins^8^,^10^. The TGF-β2–treated keratocytes treated with IGF-1 in combination with SAHA had similar levels of the corneal crystallin ALDH3A1 to the keratocytes of the negative control (Fig. 3a, IGF-1, combo SAHA; p = 0.058, p = 0.454, respectively). Furthermore, the TGF-β2–treated keratocytes treated with halofuginone alone, the IGF-1 plus halofuginone combination, and SAHA alone, all showed lower ALDH3A1 levels (Fig. 3a, HAL, combo HAL, SAHA), and were similar to the TGF-β2 positive control.

### IGF-1 and SAHA, alone and in combination, regulate the secretion of keratocan and biglycan

**Figure 4:**
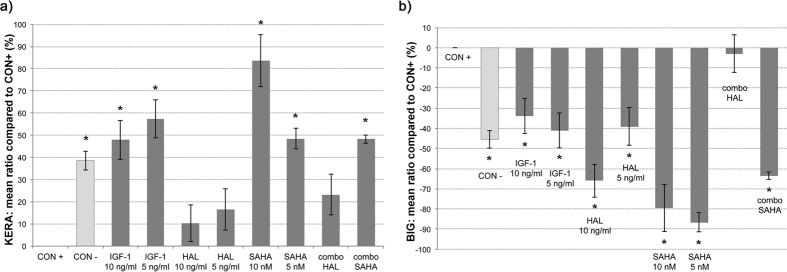
Secretion of keratocan (KERA, **a**) and biglycan (BIG, **b**), as analyzed using ELISAs. Mean relative ratios (%, ± SD) of absorbance at 590 nm in cell culture supernatants. *p < 0.001 compared to CON+ (ANOVA). For details of conditions, see legend to Fig. 1.

Compared to the TGF-β2–treated keratocytes of the positive control (Fig 4a, CON+), the keratocan levels in the culture supernatants were significantly higher for all of the further additions (Fig. 4a; p < 0.001), except for the treatments that included halofuginone (Fig. 4a, HAL, combo HAL). On the other hand, the biglycan levels were significantly reduced compared to the TGF-β2–treated keratocytes under all of these conditions (Fig. 4b, p < 0.001), except for the IGF-1 plus halofuginone combination (Fig. 4b, combo HAL). Biglycan is normally not present in the corneal stroma, and it is seen only for a disorganized ECM; i.e., in a corneal scar. However, along with other proteoglycans, keratocan is responsible for precise collagen fibril alignment in the normal cornea^7^. For the TGF-β2–treated keratocytes there was a 38.0% ± 4.3% decrease in keratocan secretion and a 45.5% ± 7.2% increase in biglycan secretion compared to the negative control. The profiles of these proteoglycans were mainly influenced by SAHA addition to these TGF-β2–treated keratocytes. Here, 10 nM and 5 nM SAHA were used alone and in the combination with 10 ng/ml IGF-1 (Fig. 4a,b, SAHA, combo SAHA), which resulted in increases for keratocan secretion of 83.7% ± 11.8%, 48.5% ± 4.7%, and 48.3% ± 1.9%, respectively, and in decreases for biglycan secretion of 79.4% ± 6.6%, 86.6% ± 1.8%, and 63.4% ± 10.6%, respectively, compared to the TGF-β2 positive control. The TGF-β2–treated keratocytes treated with SAHA in combination with IFG-1 had similar expression profiles of these proteoglycans to the negative-control keratocytes (Fig. 4a,b, combo SAHA, CON−).

### Inhibition of keratocyte transdifferentiation into myofibroblasts

**Figure 5:**
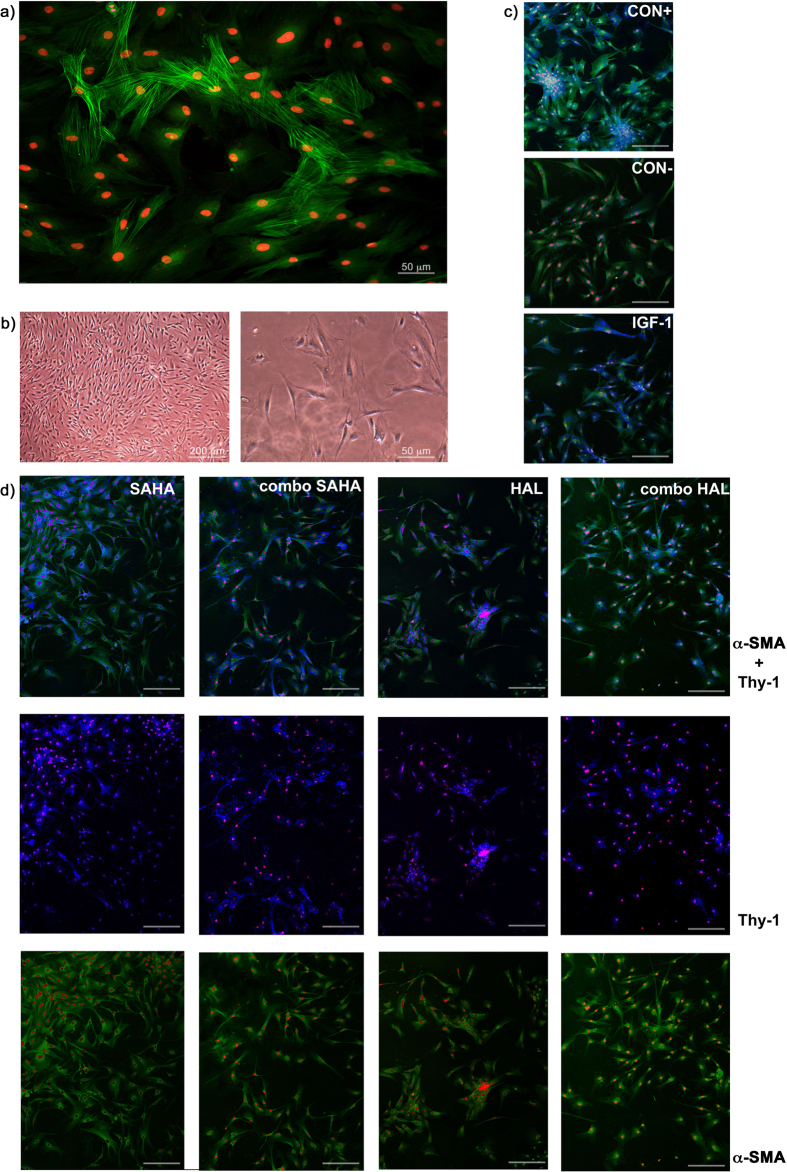
Representative microscopy images of human corneal stromal cells activated by TGF-β2 (corneal stromal wound model), and under treatments as defined in the legend to Fig. 1. **(a)** Laser scanning confocal microscopy of transdifferentiation of keratocytes to myofibroblasts after exposure to 10 ng/ml TGF-β2, showing labeling of α-SMA (green, AlexaFluor® 488) and nuclei (red, DAPI). **(b)** Phase-contrast light microscopy of isolated primary human corneal keratocytes and corneal myofibroblasts after exposure to 10 ng/ml TGF-β2. **(c**,**d)** Representative laser scanning confocal microscopy images following treatments of keratocytes as detailed in legend to Fig. 1, showing labeling of Thy-1 (blue, PE/Cy5®), α-SMA (green, FITC), and nuclei (red, DAPI). Scale bars, 100 μm. Images are representative of three independent experiments.

These human keratocyte cultures contained dendritic cells that have little cytoplasm, and that stain positive for keratocan and negative for α-SMA fibers. Under the stimulation with TGF-β2, these keratocytes transdifferentiated into myofibroblasts, with a gain in cell volume and the formation of α-SMA stress fibers (Fig. 5a,b and Supplementary Fig. S1).

We used confocal microscopy to simultaneously analyze the expression of Thy-1 and the formation of α-SMA fiber bundles, which allowed us to roughly differentiate between keratocytes, wound-type corneal fibroblasts, and corneal myofibroblasts. The TGF-β2–treated keratocytes of the positive control showed Thy-1–positive cells with α-SMA stress fibers, namely myofibroblasts (Fig. 5, CON+), while in the negative-control naïve human keratocytes there was very little Thy-1 labeling and only the diffuse presence of α-SMA in the cells (Fig. 5, CON−). This diffuse α-SMA corresponded to the observations of unstructured actin in keratocytes reported previously^52^. When SAHA or halofuginone were added to the TGF-β2–treated keratocytes in the wound model, the myofibroblastic phenotype was less common, although the cells were more spindle shaped and larger, compared to the negative control (Fig. 5, SAHA, HAL). Thy-1 was very prevalent in the TGF-β2–treated keratocytes with SAHA, and less so with the halofuginone treatments, where the α-SMA stress fibers were less prominent than in the TGF-β2–treated keratocytes of the positive control. In the presence of Thy-1, this indicates fibroblastic transdifferentiation, as opposed to myofibroblastic transdifferentiation^12^.

### The combination of IGF-1 plus halofuginone, but not SAHA, increases keratocyte proliferation

**Figure 6:**
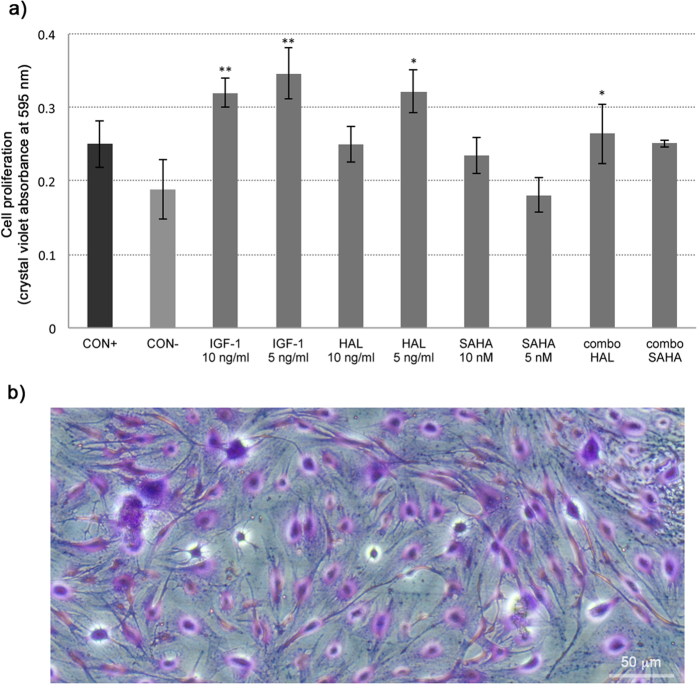
Crystal violet proliferation assay. **(a)** Proliferation rates of the keratocytes under treatments as defined in legend to Fig. 1, as revealed by crystal violet assays. *p < 0.001 compared to CON−; **p < 0.001 compared to CON+ and CON− (ANOVA). For details of conditions, see legend to Fig. 1. (**b**) Representative phase-contrast light microscopy image of keratocytes after treatment with crystal violet, before photometric measurements.

The proliferative activity of the TGF-β2–treated keratocytes was determined after 48 h of treatment, using the crystal violet assay (Fig. 6, CON+). This positive control was not significantly diminished in the keratocytes treated with SAHA and halofuginone, as also for the negative control (Fig. 6); indeed, the TGF-β2–treated keratocytes that were also treated with 5 ng/ml halofuginone alone and in combination with 10 ng/ml IGF-1 showed enhanced proliferation (Fig. 6, HAL, combo HAL). The combination of 10 nM SAHA with 10 ng/ml IGF-1 showed proliferation rates that were not significantly different from that of the TGF-β2 positive control (Fig. 6, combo SAHA, CON+). Both concentrations of IGF-1 used alone (Fig. 6, 5 ng/ml, 10 ng/ml) with the TGF-β2–treated keratocytes demonstrated significantly increased proliferation compared to both the TGF-β2 positive control and the negative control (p < 0.001), with no significant difference seen between these IGF-1 concentrations.

## Discussion

Keratocytes have a unique role in vision, as they sustain the corneal stroma and maintain corneal transparency, and they have can provide injury repair, and return the corneal stroma to its non-opaque normalcy. The loss of stromal transparency during wound healing has been linked to myofibroblast transdifferentiation, and thereby to errant ECM production and increased intracellular light scattering. The *in-vitro* data from the present study indicate that these reference antifibrotic compounds that are approved for human use (i.e., SAHA and halofuginone) can provide beneficial influence over the corneal stromal healing process, especially when combined with IGF-1. They thus favor a non-scarring healing pathway, while propagating elements of transparency.

**Figure 7:**
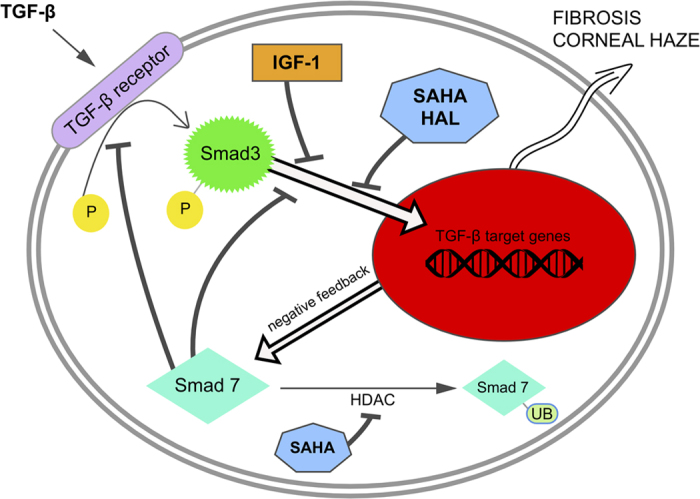
Schematic representation of the inhibitory effects of IGF-1, suberanilohydroxamic acid (SAHA) and halofuginone (HAL) on the TGF-β pathway in keratocytes. As shown with imaging flow cytometry, IGF-1, SAHA and halofuginone block Smad3 translocation to the nucleus. Inhibitory Smad7 forms as a negative-feedback loop^53^, blocking Smad3 phosphorylation and nuclearization. SAHA can also inhibit HDACs, which leads to acetylation and subsequent ubiquitination of Smad7^54^, resulting in higher intracellular Smad7 levels, as observed in the present study. P, phosphate group; UB, ubiquitin; HDAC, histone deacetylase.

The canonical Smad signaling pathway of TGF-β2 was successfully inhibited by SAHA and halofuginone in the present study. IGF-1 alone decreased Smad3 nuclearization, which is a novel finding for human keratocytes. The combination of IGF-1 with these well-established antifibrotics, SAHA and halofuginone, yielded more pronounced block of the TGF-β2–promoted translocation. Inhibition of Smad3 phosphorylation might be the main mechanism behind this effect^40^. Smad7 is an inhibitory feedback transducer, and as such, it also inhibits phosphorylation of Smad3, as has been shown in corneal wounding models^31^,^40^,^47^. According to the present study, SAHA appears to have effects through a separate mechanism, by which it causes higher levels of Smad7 even though it decreases the transcriptional activity of TGF-β2. It has been reported that proteasome-mediated degradation of Smad7 depends upon a balance between acetylation, deacetylation, and ubiquitination^53^. HDACs tend to deacetylase Smad7 and to decrease its stability, thus channeling it toward ubiquitination and degradation^54^. We suggest that as an HDAC inhibitor, SAHA disrupts the activity of specific HDACs, thereby preventing the degradation of Smad7 in the cytosol, and apparently promoting greater inhibition of fibrosis through the TGF-β/Smad pathway (see schematic representation in Fig. 7).

This study of Smad3 and Smad7 has provided relevant insight into TGF-β/Smad signaling, and it has shown that the antifibrotic compounds investigated (i.e., SAHA and halofuginone) inhibit this pathway to a significant extent. Furthermore, it has demonstrated that addition of IGF-1 inhibits translocation of Smad3 to the nucleus in its own right, with these effects potentiated when combined with SAHA (Fig. 7). This is the first study to show that IGF-1 can inhibit Smad3 translocation to the nucleus in human keratocytes. However, we did not investigate directly other intracellular pathways here, which can also propagate some of the TGF-β signals, such as mitogen-activated protein kinase^55^ and the Rho family proteins, which might take part in this system.

The imaging flow cytometry analysis performed here was applied to large numbers of cells (n = 3000), where each individual cell was taken into account. Thus, rather than using a generalization based upon population means for gene expression or proteome investigation, the events of Smad3 nuclearization in each individual cell were viewed and analyzed; i.e., using a single-cell approach. According to the literature, the present study is the first to use imaging flow cytometry for the characterization of intracellular events in keratocytes.

The IDEAS analysis software was used here to measure the fluorescence of the specific antibodies that were bound to the target proteins, with the calculation of the median fluorescence intensities, and thus the relative levels of these target proteins (see Methods). All of the keratocytes were prepared under the same conditions and were measured with identical machine calibration in a single day. The weakness of quantitative analysis of immunofluorescence using flow cytometry is the wide dispersion of the data obtained, which here were carefully weighted with the calculation of the median fluorescence intensities and the use of box and whisker plots. This approach for the examination of cell cultures at the subcellular level using imaging flow cytometry analysis as the first experimental step for the validation of a therapy was recently verified^56^,^57^.

While with flow cytometry the native shape of cells is lost, confocal fluorescence microscopy provided the proof-of-concept here, as an additional insight into the differentiation of these keratocytes. Hassell and Birk^11^ reported that wound-type fibroblasts have the most important role in regenerative wound healing *in vivo*, while myofibroblasts lead to erroneous ECM formation and scarring. In the present study, IGF-1 and the selected antifibrotics prevented this transdifferentiation to myofibroblasts, with higher proportions of Thy-1-positive wound-type fibroblasts in this cell-culture model of corneal stromal wounding, which would favor the regenerative pathway of wound healing^11^,^12^.

A crystallin (i.e., ALDH3A1) and two proteoglycans (i.e., keratocan, biglycan) were studied here, which have been shown to be representative of intracellular and ECM transparency, respectively^8^,^11^. Differences in the crystallin levels were shown under the different stimulation conditions, where the lowest levels were seen for the TGF-β2–treated keratocytes of the positive control, with a prevalence of myofibroblasts in the immunofluorescent microscopy. All of the treatments inhibited this TGF-β2–promoted depletion of ALDH3A1 in the cytosol to some extent, although significant inhibition was only achieved by IGF-1 alone and in combination with SAHA. The levels of the crystallin in the other treated keratocytes were significantly lower compared to the negative control, which confirms previous findings that the expression of ALDH3A1 is lower in transdifferentiated fibroblasts than in native keratocytes^11^,^58^. Furthermore, the keratocytes in the ELISA for the two proteoglycans saw the secretion of the highest levels of keratocan and the lowest levels of biglycan under the treatments with SAHA and IGF-1 and their combination, which indicates that under these conditions the ECM should appear more transparent *in vivo*.

The secretion of keratocan was significantly increased by IGF-1 alone, which has also been reported for lumican^13^, both of which are significant structural proteoglycans in terms of a transparent collagenous ECM. Furthermore, IGF-1 promoted a beneficial increase in cytosolic ALDH3A1, which to the best of our knowledge has not been reported in previous studies. The decreased TGF-β2–promoted Smad3 nuclearization that was seen here for IGF-1 was previously suggested in studies of muscle fibrosis^59^,^60^ and prostate cancer^61^, but to date this has not been shown for human keratocytes. Similar to previous *in-vitro* studies in corneal wound healing^51^, the confocal microscopy in the present study indicated that TGF-β2–promoted transdifferentiation to myofibroblasts is less likely to occur with the addition of IGF-1. As demonstrated by the proliferation assays here, the TGF-β2–promoted proliferation rates under IGF-1 treatment of the keratocytes was significantly increased, which corresponds to a report of exogenous IGF use on human corneal fibroblasts^51^. We can deduce here that IGF-1 alone can decrease the probability of corneal stromal scar formation, while exerting its well-known positive effects on keratocyte proliferation and wound repair. Hence, IGF-1 is not only a valid aid to inhibitors of the TGF-β/Smad pathway in keratocytes, but it can act alone as a potent modulator of fibrosis.

Although our study indicates the need for further investigations and *in-vivo* validation to confirm these data, it appears that IGF-1 supplemented with SAHA and halofuginone can be advocated for anti-fibrotic therapy of corneal wounding.

## Methods

### Isolation and cultivation of primary human corneal keratocytes

The human keratocytes used in this study were isolated and cultured in serum-free medium according to a modified procedure described by Jester *et al*.^62^ and Pei *et al*.^10^. Briefly, corneal tissue was harvested from donor corneas that are normally discarded during the standard surgical procedure for perforative keratoplasty. Written informed consent for perforative keratoplasty was obtained. The Slovenian National Ethics Committee approved the collection of these tissue samples (No. 132/11/13, 20.12.2013), furthermore, our study was done in accordance with the Declaration of Helsinki and the Oviedo Convention. The corneal epithelium and Descemet’s membrane with the endothelium were scraped off using a hockey knife, while the remaining stroma was stored in sterile advanced Dulbecco’s modified Eagle’s medium (DMEM; Life Technologies Ltd, Paisley, UK). The tissue was cut into blocks and incubated at 37 °C overnight in 2.0 mg/ml collagenase type I (Sigma-Aldrich, Grand Island, USA) diluted in DMEM. The digested material was collected and centrifuged in a 50-ml tube at 200 × *g* for 5 min, to obtain the pellet of primary human keratocytes. Some of these cell pellets were frozen in liquid nitrogen and stored at −80 °C until analysis. These isolated cells were washed in DMEM and cultured under serum-free conditions with the addition of 100 U/ml penicillin, 1 mg/ml streptomycin, and 2 mM L-glutamine. The cells were resuspended and transferred into collagen-coated tissue culture flasks for further cultivation at 37 °C and in an atmosphere of 5% CO_2_. After 48 h, the non-adherent cells were removed and the adherent cells were cultured further. At the time of the experiments, these human keratocytes had reached 3–6 passages.

### TGF-β2 activation and treatment with IGF-1 and the antifibrotics

The human keratocytes were cultured in serum-free medium as described above (with the negative control as the untreated human keratocytes). First, the upper concentrations of the reference bioactives were set according to the 3-(4,5-dimethylthiazol-2-yl)-2,5-diphenyltetrazolium bromide (MTT) cell-viability assay (see Supplementary Fig. 2). A reductionist corneal stromal wounding model was initiated by addition of 10 ng/ml TGF-β2^15^ (R&D systems, Minneapolis, USA) into the DMEM for 48 h (as the positive control). These ‘activated keratocytes’ were also treated with the different bioactives, which were added at the time of the ‘wounding’, with parallel incubation of the wound model for 48 h, due to the fast transient nature of these keratocytes. The bioactives used were: 10 ng/ml, 5 ng/ml recombinant IGF-1 (R&D systems, Minneapolis, USA), 10 ng/ml, 5 ng/ml halofuginone (Santa Cruz Biotechnology, Heidelberg, Germany), and 10 nM, 5 nM SAHA (Tocris Bioscience, Bristol, UK). The 10 ng/ml IGF-1 was also tested in combination with one concentration of each of the antifibrotics: 10 ng/ml halofuginone, and 10 nM SAHA. After completion of the first proliferative assays, the concentrations of halofuginone and SAHA used for the further experiments were chosen as 5 ng/ml and 10 nM, respectively. These human keratocytes were also used for further testing, as described below.

### Analysis of the TGF-β/Smad signaling pathway using imaging flow cytometry

To quantitatively determine Smad7 expression and the rate of Smad3 nuclear translocation, these human keratocytes were analyzed using multispectral imaging flow cytometry (ImageStreamX; Amnis Coorporation, Seattle, USA). The keratocytes were harvested, followed by their permeabilization, with the following antibodies used for the staining, according to manufacturer protocols: mouse anti-Smad7 or rabbit anti-Smad3 antibodies, with Cy5 goat anti-mouse or goat AlexaFluor® 488 anti-rabbit secondary antibodies (Abcam, Cambridge, UK), and 7AAD (BD Pharminogen) to visualize the nucleus. Cell images were acquired for 3000 events per sample at 40x magnification, using 488 nm and 658 nm lasers lines, with the fluorescence collected using two spectral detection channels. For double-stained keratocytes, two single-stained controls were used to compensate for the fluorescence between the channel images. The cell images were analyzed using the IDEAS image-analysis software (Amnis). First, cells within the focal plane were selected using a plot of the image contrast *versus* the root-mean-squared gradient. Then the aspect ratio *versus* the cell area was gated to isolate populations of single cells on a bivariate plot^57^. The Smad7 probe median fluorescence intensities were determined for the relative intracellular quantification. The Smad3 nuclear translocation was calculated with a similarity algorithm (similarity dilate), where the software compared the location of the Smad3 *versus* the location of the nucleus. The subpopulation of keratocytes in which Smad3 translocation occurred were calculated and expressed as percentages^56^.

### Measurement of intracellular ALDH3A1 using imaging flow cytometry

The human keratocytes were harvested, permeabilized, and treated with a rabbit anti-ALDH3A1 antibody labeled with a AlexaFluor® 488 goat anti-rabbit antibody (Abcam), according to the manufacturer protocols. 7AAD (BD Pharminogen, Heidelberg, Germany) was used for nuclear staining. The keratocyte images were obtained using imaging flow cytometry and the IDEAS software, as described above. For the relative quantification, the median fluorescence intensities of ALDH3A1 were determined.

### Evaluation of keratocan and biglycan secretion using enzyme-linked immunosorbent assays

The human keratocytes were seeded at 10,000 cells/well in 96-well plates. They were treated as described above, and after 48 h the levels of keratocan and biglycan in the supernatants were determined using ELISA kits (Invitrogen Co., Camarillo, USA, and USC Life Science Inc., Wuhan, China), based on the manufacturer protocols. All of these assays were performed in triplicate.

### Confocal fluorescence microscopy

The human keratocytes were immunostained with antibodies against Thy-1 and α-SMA, as previously described^12^. Briefly, the keratocytes were grown on collagen-coated 8-well glass chamber slides for 24 h until the respective treatments, as described above. After 48 h, they were fixed in 2% paraformaldehyde in phosphate-buffered saline (pH 7.4). They were then permeabilized and reacted with a PE/Cy5-conjugated anti-human Thy-1 antibody (Abcam) and a FITC-conjugated anti-human α-SMA antibody (Abcam), according to manufacturer protocols. The samples were counter-stained for the nucleus, and mounted with Fluoroshield^TM^ with DAPI (Sigma-Aldrich). These keratocytes were then evaluated using laser-scanning confocal microscopy (Leica TCS SP5 II; Leica Microsystems, Mannheim, Germany).

### Cell proliferation

To perform the cell proliferation assays, the human keratocytes were seeded at 10,000 cells/well in 96-well plates, at a concentration of 30 viable cells per well. After adding TGF-β2 and the various treatments, as described above, the cells were stained with crystal violet and the absorbance was measured at 595 nm. All of these cell proliferation assays were carried out in triplicate.

### Statistical analysis

The data were collected and analyzed using Statistical Package for Social Sciences (SPSS), version 16. The data are expressed as the means ± SD for the parametric tests, and median ± interquartile range for non-parametric tests. Data normality was analyzed using Shapiro-Wilk tests. Comparison of groups were performed using one-way ANOVA for multiple comparisons, followed by Holm-Sidak *post-hoc* analysis or Mann-Whitney rank sum tests for comparisons of medians. Chi^2^ tests were used to analyze the significance of the Smad3 nuclear translocation. The differences were considered statistically significant for p < 0.001, with a 95% confidence interval.

## Additional Information

**How to cite this article**: Sarenac, T. *et al*. Single-cell analysis reveals IGF-1 potentiation of inhibition of the TGF-β/Smad pathway of fibrosis in human keratocytes *in vitro*. *Sci. Rep*. **6**, 34373; doi: 10.1038/srep34373 (2016).

## Supplementary Material

Supplementary Information
