# Supplementary material for: Single-cell analysis reveals IGF-1 potentiation of inhibition of the TGF-β/Smad pathway of fibrosis in human keratocytes in vitro
Source: Sci Rep. 2016 Sep 30;6:34373. doi: 10.1038/srep34373 (PMC5043377; doi:10.1038/srep34373)
Supplement: Supplementary Information [file srep34373-s1.pdf]

Single-cell analysis reveals IGF-1 potentiation of inhibition of the TGF-β/ Smad pathway of fibrosis in human keratocytes *in vitro*

Tomislav Sarenac, Martin Trapecar*, Lidija Gradisnik, Marjan Slak Rupnik, Dusica Pahor

* martin.trapecar@gladstone.ucsf.edu

SUPPLEMENTARY FIGURES

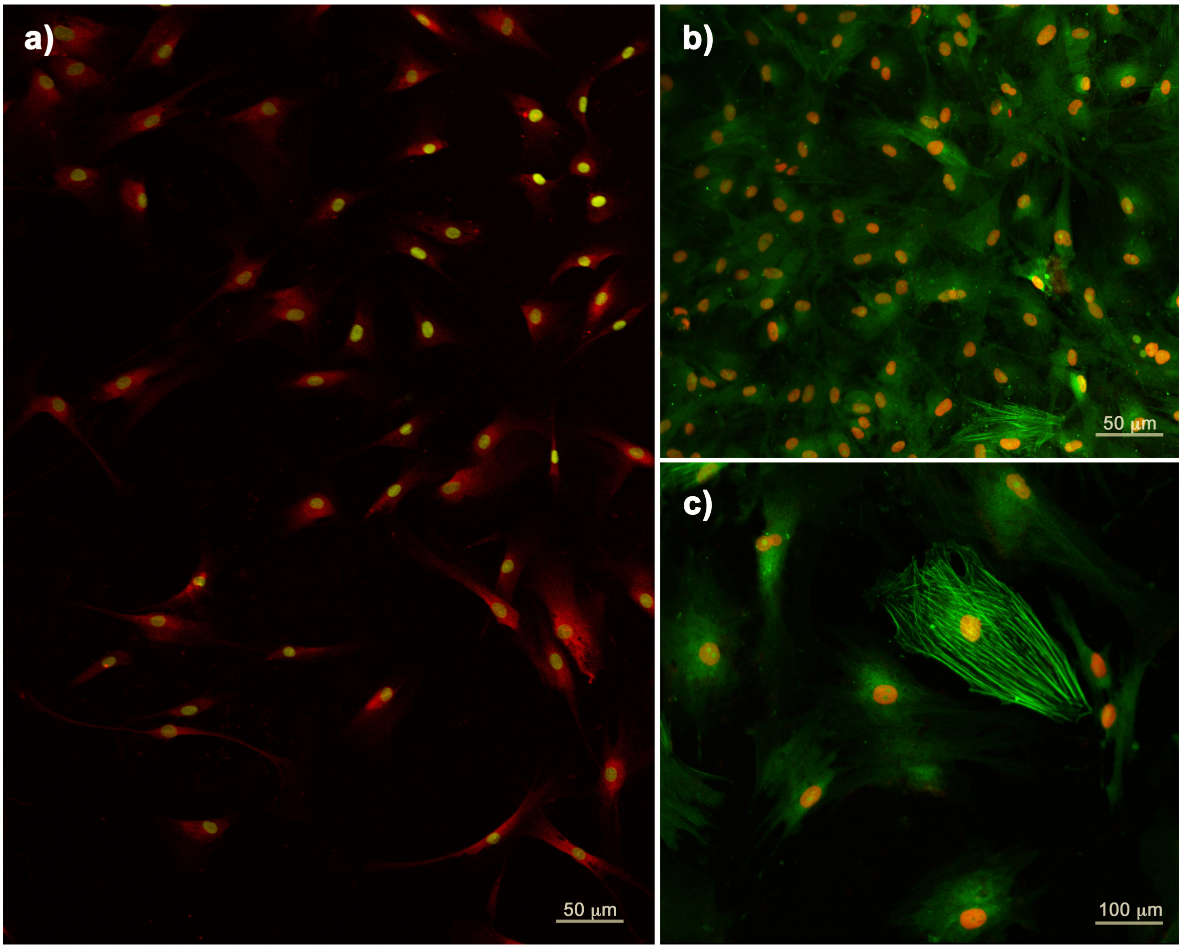

Supplementary Figure S1. Representative confocal microscopy images of isolated primary human corneal keratocytes in culture.

(a, b) Human keratocytes were labelled for the specific proteoglycan keratocan (red, AlexaFluor® 488), and the nuclei (green, DAPI) (a) and for α-SMA (green, FITC) and the nuclei (red, DAPI) (b). These keratocytes showed no α-SMA fibril formation, except for some cells at the edges of the microscopy field. **(c)** A differentiated corneal myofibroblast with α-SMA fibres (green, FITC; nuclei, red, DAPI) surrounded by keratocytes that show no intracellular α-SMA fibril formation and are smaller in size.

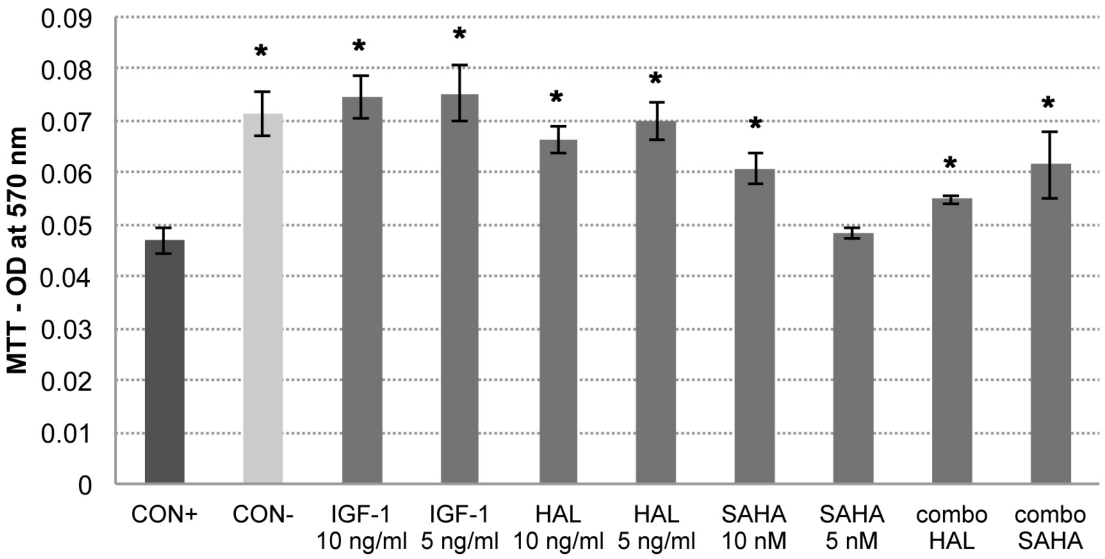

Supplementary Figure S2. MTT cell viability assays of keratocytes following treatments.

*, $p < 0.001$ compared to CON+ (ANOVA). CON+, positive control of 10 ng/ml TGF-β2; CON-, negative control for naïve human keratocytes. All of the following conditions also included 10 ng/ml TGF-β2: 10 ng/ml, 5 ng/ml IGF-1; 10 ng/ml, 5 ng/ml halofuginone (HAL); 10 ng/ml IGF-1 plus 5 ng/ml halofuginone (combo HAL); 5 nM, 10 nM SAHA; 10 ng/ml IGF-1 plus 10 nM SAHA (combo SAHA).
